# Development and validation of a novel questionnaire to describe and assess sensations and triggers associated with refractory and unexplained chronic cough

**DOI:** 10.1136/bmjresp-2024-002430

**Published:** 2024-08-13

**Authors:** Shannon Galgani, Chelsea Sawyer, Jenny King, Rachel Dockry, James Wingfield-Digby, Kimberly Holt, Joanne Mitchell, Shilpi Sen, Danielle Birchall, Francesca Solari, Jacky Smith, Janelle Yorke

**Affiliations:** 1 Cough Research Team, Manchester University NHS Foundation Trust, Manchester, UK; 2 Division of Psychology and Mental Health, The University of Manchester, Manchester, UK; 3 Division of Infection, Immunity and Respiratory Medicine, The University of Manchester Faculty of Biology Medicine and Health, Manchester, UK; 4 Simplifying Science, Warrington, UK; 5 Trauma and Orthopaedics, Ysbyty Gwynedd, Bangor, UK; 6 School of Nursing, The Hong Kong Polytechnic University, Hong Kong, Hong Kong

**Keywords:** Cough/Mechanisms/Pharmacology, Surveys and Questionnaires

## Abstract

**Introduction:**

Refractory or unexplained chronic cough (RUCC) is a common clinical problem with no effective diagnostic tools. The Sensations and Triggers Provoking Cough questionnaire (TOPIC) was developed to characterise cough in RUCC versus cough in other conditions.

**Methods:**

Content analysis of participant interviews discussing the sensations and triggers of chronic cough informed TOPIC development. Participants with chronic cough completed the draft-TOPIC (a subset repeating 5–7 days later), St George’s Respiratory Questionnaire (SGRQ), Cough Severity Diary (CSD) and Global Rating of Change Scale. The draft-TOPIC item list was reduced in hierarchical and Rasch analysis to refine the questionnaire to the TOPIC.

**Results:**

49 items describing the triggers and sensations of cough were generated from participant interviews (RUCC n=14, chronic obstructive pulmonary disease (COPD) n=11, interstitial lung disease (ILD) n=10, asthma n=11, bronchiectasis n=3, cystic fibrosis n=7). 140 participants (median age 60.0 (19.0–88.0), female 56.4%; RUCC n=39, ILD n=38, asthma n=45, COPD n=6, bronchiectasis n=12) completed draft-TOPIC, where items with poor ‘fit’ for RUCC were removed to create TOPIC (8 trigger items, 7 sensation items). Median TOPIC score was significantly higher in RUCC (37.0) vs ILD (24.5, p=0.009) and asthma (7.0, p<0.001), but not bronchiectasis (20.0, p=0.318) or COPD (18.5, p=0.238), likely due to small sample sizes. The Rasch model demonstrated excellent fit in RUCC (χ^2^=22.04, p=0.85; PSI=0.88); as expected. When all participant groups were included, fit was no longer demonstrated (χ^2^=66.43, p=0.0001, PSI=0.89) due to the increased heterogeneity (CI=0.077). TOPIC correlated positively with SGRQ (r=0.47, p<0.001) and CSD (r=0.63, p<0.001). The test–retest reliability of TOPIC (intraclass correlation coefficient) was excellent (r=0.90, p<0.001).

**Conclusions:**

High TOPIC scores in the RUCC patients suggest their cough is characterised by specific sensations and triggers. Validation of TOPIC in cough clinics may demonstrate value as an aid to identify features of RUCC versus cough in other conditions.

**WHAT IS ALREADY KNOWN ON THIS TOPIC**

- Refractory or unexplained chronic cough (RUCC) is a common clinical problem. However, there are currently no effective diagnostic tools, and patients often undergo numerous investigations and treatment trials prior to diagnosis.

**WHAT THIS STUDY ADDS**

- Here, we describe the development of The Sensations and Triggers Provoking Cough (TOPIC) questionnaire which aimed to characterise the triggers and sensations of cough experienced by patients with RUCC, compared with cough secondary to other respiratory diseases.

**HOW THIS STUDY MIGHT AFFECT RESEARCH, PRACTICE OR POLICY**

- Following validation, the TOPIC questionnaire may aid early identification of patients with features of RUCC so that they can access appropriate services and treatments.

## Introduction

 Cough is an important protective mechanism, yet it is also one of the most common complaints for which medical advice is sought.^1^ Chronic cough—defined as cough persisting for more than 8 weeks—has a global prevalence of 9.6% and has a substantial impact on quality of life (QOL).^2^,^4^

While chronic cough is a recognised symptom of multiple other conditions (eg, asthma, gastro-oesophageal reflux disease, chronic obstructive pulmonary disease (COPD) and bronchiectasis), 59% of patients referred to specialist cough clinics have cough that persists despite treatment of the underlying condition, or cough of an unknown aetiology termed refractory, and unexplained chronic cough, respectively (RUCC).^5^,^7^ Despite this, management of RUCC remains a challenge with many patients undergoing numerous investigations and treatment trials for multiple conditions prior to diagnosis, perhaps in part due to the diagnosis of RUCC being one of exclusion.^8^,^11^

Evidence supports hyperexcitability of the neuronal pathways controlling cough, termed cough hypersensitivity, to underly presentations of chronic cough in which cough is evoked by exposure to usually innocuous stimuli.^10^ Patients with RUCC often report somatic sensations and triggers associated with their cough such as irritation and tickling in the throat, urge to cough (UTC) and sensitivity to allotussive triggers (eg, phonation, food and olfactory triggers)^12^,^17^; yet clinically recommended QOL and cough symptom questionnaires do not effectively encompass these sensations and triggers.^6^

In recent years, some tools designed to measure the somatic sensations associated with cough hypersensitivity have emerged. However, their use in RUCC is limited due to a lack of comparison groups (Newcastle Laryngeal Hypersensitivity Questionnaire^18^; Hull Airway Reflux Questionnaire (HARQ)),^19^ reporting only cough sensations and triggers as a binary yes/no response (thus omitting quality and frequency; Cough Hypersensitivity Questionnaire),^14 15 20^ or reporting non-specific cough sensations and triggers (McMaster Cough Severity Questionnaire).^16 17 21^ To be useful in identifying cough in RUCC, work is therefore, needed to effectively characterise cough triggers and sensations as they present in RUCC compared with those of other conditions causing cough.

Here, we describe the development of The Sensations and Triggers Provoking Cough questionnaire (TOPIC), to characterise the triggers and sensations of cough experienced by patients with RUCC compared with cough in other lung conditions.

## Methods

### Study design and patient involvement

An exploratory sequential design consisting of three stages was used, with patient involvement central to stages I and II. Stage I (item generation) and stage II (cognitive debriefing) informed the questionnaire’s draft item list from participant interviews and focus group discussions. A cross-sectional quantitative design was used for stage III (item reduction and psychometric analyses) where following participant completion of the draft questionnaire, items were reduced using hierarchical and Rasch analysis (methodology previously described^22^). Identifying core descriptors of cough and their association with different respiratory pathologies enabled the development of a questionnaire that captures the triggers and sensations experienced most in RUCC versus other chronic cough aetiologies.

For all stages, participants (>18 years) with a persistent cough and physician diagnosis of RUCC (cough >8 weeks, normal lung function and chest X-ray), COPD, interstitial lung disease (ILD), asthma, cystic fibrosis or non-cystic fibrosis bronchiectasis were recruited. The RUCC cohort was defined as participants with cough as a persistent problem, despite adequate control of other identifiable conditions that could be associated with cough (eg, gastro-oesophageal reflux disease, asthma, upper airways disease), or cough as a persistent problem despite no other identifiable conditions. This differed from the participants in the other cohorts, as while they had a persistent cough, it was not a major symptomatic complaint.

Participants were recruited from community services (pulmonary rehabilitation and asthma services), specialist secondary and tertiary respiratory clinics and an existing research database of patients who previously indicated interest in taking part in research (The Manchester Allergy, Respiratory & Thoracic Surgery Biobank (10-H1010-7)), in Manchester, UK.

Participants with cystic fibrosis were included in stage I only as focus group discussions with this patient group are discouraged due to infection risk, and difficulty accessing these patients during the COVID-19 pandemic. Subsequently, non-cystic fibrosis bronchiectasis patients were included in stages II and III.

Participants were excluded if they had a recent (<4 weeks) upper respiratory tract infection, were taking ACE inhibitor or cough suppressant treatment or could not understand written English or provide informed consent. Stages I and II excluded active smokers.

STROBE (strengthening the reporting of observational studies in epidemiology cross-sectional reporting) guidelines were used.^23^

### Stage I: initial item generation

Participants from respiratory clinics were invited to attend one-to-one semi-structured interviews in a private room or over the telephone. Each participant (n=4–7 per diagnostic group) was asked a series of exploratory questions regarding their cough from the TOPIC interview guide (online supplemental figure 1S) to elicit information regarding their experience, sensations and triggers of cough. The interview guide was developed following a literature review of studies exploring people’s experience and descriptions of cough and incorporating the experience of clinicians delivering local cough services.^24 25^ The interviews were audio recorded if the participant agreed, or handwritten notes were made throughout to document key information and cough descriptors. Data from this stage have previously been reported in conference abstract.^26^

### Stage II: cognitive debriefing

Participants were invited to a 60 minute focus group discussion (n<10 per group, split by diagnosis). The most frequent descriptors of cough generated from stage I (online supplemental table 1S) were presented in dual-moderated discussions (one moderator running the session, the other ensuring topic coverage), asking participants to describe what each word meant to them, the image it conjured up and whether they could identify the most appropriate word. TOPIC draft items were generated from content analysis of the interview transcripts, with the most agreed on and articulate descriptors of cough identified by the participants being selected (online supplemental table 2S).

### Stage III: item reduction

Participants were asked to complete the draft TOPIC questionnaire (online supplemental table 2S) in person during their routine clinical appointment or via post. The draft TOPIC questionnaire comprised two subscales of 31 trigger and 18 sensation items (n=49), scored on a 6-point Likert-type scale (0 (never), 1 (a little of the time), 2 (some of the time), 3 (a lot of the time), 4 (most of the time) and 5 (always)). Subject demographics, medical history, current medications, duration of cough and most recent lung function test (forced expiratory volume in 1 second, forced vital capacity, FEV1/FVC ratio) were recorded.

Up to 50 participants were recruited for each cohort, with at least 10 participants per factor of interest required to perform factor analysis.^27^ Up to 90 participants (the first willing 15 participants per cohort) were asked to complete the TOPIC questionnaire a second time 5–7 days later to determine test–retest reliability. These participants also completed a Global Rating of Change Scale (GroC)—a self-reported instrument designed to quantify whether a patient’s symptoms have improved, deteriorated, or remained the same over time—to assess any change in clinical status between TOPIC questionnaire completion.

To gain insight on any commonly missed items, all returned questionnaires—including those that were incomplete—were included in item reduction. However, only complete questionnaires were included when analysing the total questionnaire score.

Participants also completed the following additional questionnaires to allow construct validity testing:

St. George’s Respiratory Questionnaire (SGRQ), a self-reported, disease-specific instrument designed to measure impact on overall health, daily life and perceived well-being in participants with obstructive airways disease. SGRQ scores range from 0 to 100, with higher scores indicating more limitations.^28 29^

Cough Severity Diary (CSD), a 7-item daily diary answered on a scale of 0–10 (never–constantly), used to assess cough severity in clinical trials.^30 31^

Data from this stage have previously been reported in conference abstracts.^32 33^

### Data analysis

In stages I and II, simple manifest content analysis of participant interviews was used to derive key cough descriptors.^34^ In stage I, the most frequently observed words or phrases across eight coding units (triggers, sensation, sputum, emotion, location, frequency, time and relief) were identified by one coder and then independently checked by two others as key cough descriptors for cognitive debrief (online supplemental table 1S). In stage II, participants were asked to evaluate these key cough descriptors in diagnosis-specific focus group discussions to gain an understanding of the language participants felt accurately represented each descriptor. Participant statements regarding each descriptor were collated, resulting in identification of 49 most agreed on and articulate descriptors of cough across the sensation and trigger themes. These items constituted the draft TOPIC questionnaire (online supplemental table 2S).

In stage III, a cross-sectional description research design was developed for up to 300 participants (50 for each diagnostic group^27 35^). Hierarchical methods were applied to identify those draft TOPIC items deemed to have little discriminatory effect, which were then removed. This included flagging items with a floor (set at ≥50% selected 0 or 1) or ceiling (set at ≥50% selected 4 or 5) effect. Kruskal-Wallis tests were performed to determine if there was any diagnosis bias and Mann Whitney U-tests were performed to determine if there was any gender bias. Spearman’s correlation tests were performed to assess relationships between individual items and item-total score for the draft TOPIC questionnaire total and subscale scores and to determine any age bias.

The remaining triggers and sensations were further analysed using Rasch analysis. Items with poor fit to the Rasch model for participants with RUCC were removed (online supplemental tables 3S and 4S). A principal components analysis (PCA) using varimax rotation was used to assess the underlying structure of the final item set, where a standardised factor loading of 0.5 was used.^36 37^ Eigenvalues were used to determine the number of components extracted, with item allocation onto a component determined by a factor loading which by convention is set at >0.5 (online supplemental table 5S). This resulted in the refined 15-item TOPIC questionnaire (items listed in table 2).

To test internal reliability, the Cronbach’s alpha coefficient was calculated for the questionnaire and any subscales, where α≥0.70 demonstrated acceptable internal consistency. Reliability over time (5–7 days period) was assessed using intraclass correlation coefficients in participants who reported no perceived change in their symptoms (GRoC score ‘3: About the same’). To examine concurrent validity of the questionnaire, Spearman’s rank correlations were used to test associations between TOPIC scores, and the SGRQ and CSD. Kruskal-Wallis tests were used to compare TOPIC scores between subgroups (eg, different diagnostic groups) with Bonferroni corrections applied for multiple testing. ROC curve analysis was used to assess sensitivity and specificity of TOPIC in identifying RUCC versus non-RUCC participants. Statistical analyses were conducted by using IBM Statistical Package for Social Sciences (SPSS) V.25 or RUMM2030.

## Results

**Figure 1:**
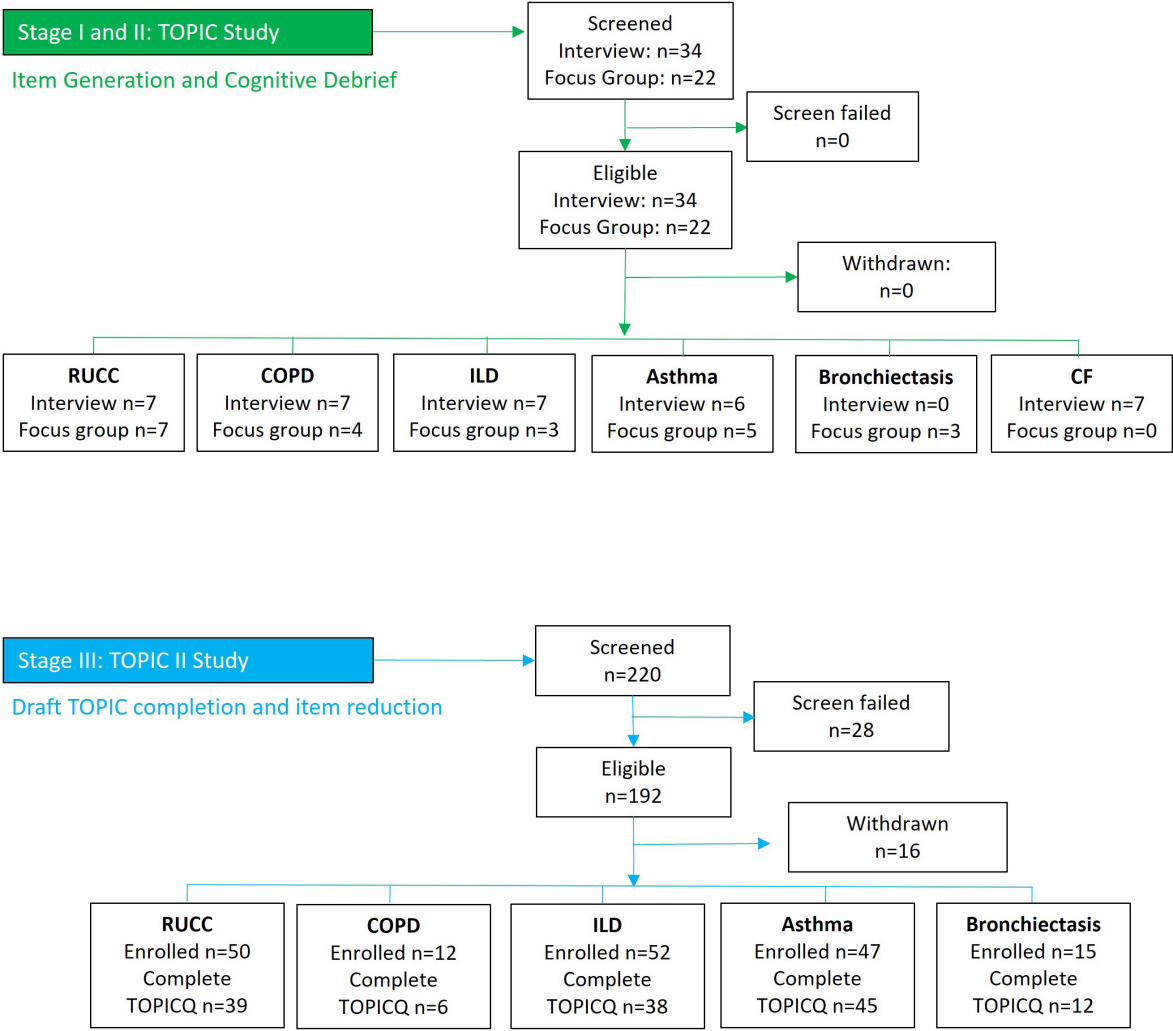
CONSORT flow diagram. Stage I: Initial item generation through content analysis of cough descriptors in 1-2-1 interviews. Stage II: Cognitive debrief of cough descriptors in focus group discussions, split by diagnosis. The most articulate and agreed on items comprised the draft TOPIC questionnaire. Stage III: Participants from respiratory cough clinics completed the draft-TOPIC questionnaire. CF, cystic fibrosis; CONSORT, consolidated standards of reporting trials*;* COPD, chronic obstructive pulmonary disease; ILD, interstitial lung disease; RUCC, refractory/unexplained chronic cough; TOPIC, the sensations and triggers provoking cough.

Figure 1 presents recruitment of participants to each stage of development. Table 1 presents the demographics of all participants recruited for each study stage. Age (median, years: stage I, 63.50; stage II, 66.5; stage III, 60.0) and sex (female: stage I, 47.1%; stage II, 45.5%; stage III, 56.4%) were similar across stages. There were no current smokers in stage I, but a small number in stage III (4.3%, n=6) half of which were in the COPD cohort (n=3). Disease severity was moderate in the stage III COPD cohort (median (IQR) FEV1 57.1% predicted (50.0–88.0)).

**Table 1:** Participant demographics

Stage I: TOPIC study interviews						
	Total group(N=34)	RUCC(n=7)	ILD(n=7)	Asthma(n=6)	COPD(n=7)	Cystic fibrosis(n=7)
Age, years(median, (range))	63.5(20–86)	66.0(39–69)	72.0(57–78)	59.0(42–65)	71.0(62–86)	23.0(20–39)
Female %	47.1	57.1	42.9	83.3	28.6	28.6
Smoking status % (n)						
Never	55.9 (19)	71.4 (5)	14.2 (1)	83.3 (5)	14.3 (1)	100.0 (7)
Ex-smoker	44.1 (15)	28.6 (2)	85.7 (6)	16.7 (1)	85.7 (6)	0.0 (0)

Stage II: TOPIC study focus groups						
	Total group(n=22)	RUCC(n=7)	ILD(n=3)	Asthma(n=5)	COPD(n=4)	Bronchiectasis(n=3)
Age, years(Median, (range))	66.5(53–80)	69.0(57–80)	71.0(65–73)	66.0(60–70)	66.5(66–73)	53.0(53–77)
Female %	45.5	57.1	0.0	40.0	50.0	66.7

Stage III: TOPIC II study						
	Total group(N=140)	RUCC(n=39)	ILD(n=38)	Asthma(n=45)	COPD(n=6)	Bronchiectasis(n=12)
Age, years(Median, (range))	60.0(19–88)	58.0(26–88)	67.0(52–83)	34.0(19–81)	61.0(53–68)	69.0(59–80)
Female %	56.4	71.8	39.5	57.8	16.7	75.0
Race, % (n)						
White	85.7 (120)	89.7 (35)	94.7 (36)	68.9 (31)	100.0 (6)	100.0 (12)
Asian	12.1 (17)	5.1 (2)	5.3 (2)	13 (28.9)	0 (0)	0 (0)
Black	1.4 (2)	5.1 (2)	0 (0)	0 (0)	0 (0)	0 (0)
Other	0.7 (1)	0 (0)	0 (0)	1 (2.2)	0 (0)	0 (0)
Smoking Status % (n)						
Never	70.0 (98)	66.7 (26)	63.2 (24)	86.7 (39)	0.0 (0)	75.0 (9)
Ex-smoker	25.7 (36)	30.8 (12)	34.2 (13)	11.1 (5)	50.0 (3)	25.0 (3)
Current smoker	4.3 (6)	2.6 (1)	2.6 (1)	2.2 (1)	50.0 (3)	0.0 (0)
Cough duration, years (median, (range))	5.7(0.4–69.8)	10.0(1.5–69.8)	4.0(0.5–20.5)	5.0(1.0–39.9)	4.7(0.5–16.0)	5.0(0.4–24.9)
FEV1 %(Median (range))	82.0(37–143)	101.0(63.0–143.0)	75.0(43.3–102.0)	89.5(37.0–120.0)	57.0(50.0–88.0)	94.0(43.0–118.0)

No clinical characteristics were collected for Sstage II. Stage III demographics were presented from participants thatwho fully completed the TOPIC questionnaire n=140/176. FEV1, forced expiratory volume in 1 second

COPDchronic obstructive pulmonary diseaseILDinterstitial lung diseaseRUCCrefractory/unexplained chronic coughTOPICThe Sensations and Triggers Provoking Cough

### Stage I: item generation

One-to-one, audio recorded interviews were conducted with 34 participants (median age 63.50 years (range 20–86)) across 5 respiratory groups (RUCC n=7, asthma n=6, COPD n=7, ILD n=7, cystic fibrosis, n=7). Age was similar across cohorts, except the cystic fibrosis participants who were younger (median age 23 years (range 20–39)). The asthma cohort was predominantly female (83.3%), whereas COPD and cystic fibrosis cohorts were predominantly male (71.4% in both groups). All groups described a ‘tickle’ or an ‘irritation’ sensation, predominantly felt in the throat and upper chest. Cystic fibrosis and COPD participants most described feeling a ‘need to clear’ their airways. The ILD group mostly described a ‘dry’, ‘tickly’ sensation. RUCC and asthma groups used a heterogeneous set of terms regarding the sensations provoking their cough. Common across groups was a hypersensitive response to things that may trigger their UTC, which coughing relieved. UTC was generally associated with negative emotions, except for some participants who described positive emotions associated with the need to clear their airways.

Content analysis of the most frequently observed words or phrases from the audio transcripts identified 18 key descriptors of cough across three themes: sensations (n=5), triggers (n=5) and secretions (n=8) (online supplemental table 1S).

### Stage II: cognitive debriefing

22 participants (median age 66.5 years (range 53–80)) were enrolled across 5 respiratory groups to take part in focus groups, split by diagnosis (RUCC n=7, COPD n=4, ILD n=3, asthma n=5, bronchiectasis n=3). Generally, there was an equal sex ratio across groups, except the bronchiectasis and asthma cohorts which were predominantly female (66.7%) and male (60.0%), respectively. Two participants had previously participated in stage I (asthma n=1, RUCC n=1). A total of 338 statements regarding the 18 key descriptors of cough were identified from the focus group transcripts. All groups favoured the terms ‘phlegm’ over ‘sputum’, ‘irritation’ over ‘tickle’ and ‘need to cough’ over ‘UTC’. All groups except asthma reported certain smells and odours as triggers. While all groups apart from COPD recognised eating as a trigger, the RUCC and asthma groups most prominently described the process of eating, swallowing certain foods and speaking as triggers. All groups related to the word crackle, apart from the RUCC group. Following the process of item refinement from cognitive interviews, 49 items were included in the draft TOPIC and applied in the next study phase (online supplemental table 2S).

### Stage III: item reduction

176 participants (median age 62.0 (range 19.0–88.0), female 56.3%, median cough duration 5.0 years (range 0.3–69.8)) were enrolled across 5 respiratory groups (RUCC n=50, ILD n=52, asthma n=47, COPD n=12, bronchiectasis n=15) to complete the 49-item draft TOPIC questionnaire (trigger items n=31, sensation items, n=18). 140 participants completed the draft TOPIC questionnaire (n=39 RUCC, n=38 ILD, n=45 asthma, n=6 COPD, n=12 bronchiectasis), with 36 participants (20.5%) returning incomplete questionnaires. TOPIC items were reduced in hierarchical and Rasch analysis.

13 trigger items and 5 sensation items were removed in hierarchical analysis. Items were removed where RUCC participants demonstrated a floor effect (>50% RUCC scored 0 or 1), and non-RUCC participants had a normal frequency, or where both RUCC and non-RUCC participants demonstrated a ceiling effect (>50% non-RUCC scored 4 or 5). 10 trigger items and 6 sensation items were then removed in Rasch analysis due to poor fit to the RUCC cohort (online supplemental table 3S and 4S).

The final eight-item trigger and seven-item sensation subscales demonstrated good fit to Rasch for all participant groups (trigger, χ^2^=9.18, p=0.91; PSI=0.77; sensation, χ^2^=19.11, p=0.16; PSI=0.84). When only participants with RUCC were entered into the model only the sensation subscale demonstrated a good fit (sensation, χ^2^=10.11, p=0.75; PSI=0.80; trigger, χ^2^=49.35, p<0.001; PSI=0.75). The final 15-item questionnaire (items listed in table 2) demonstrated an excellent fit to Rasch (χ^2^=22.04, p=0.85; PSI=0.88) for participants with RUCC. When participants from all diagnostic groups were included in the Rasch model for all 15 items the χ^2^ statistic was non-significant (χ^2^=66.43, p<0.0001, PSI=0.89), due to expected issues with heterogeneity (CI=0.077). This indicated that the underlying construct of the questionnaire behaved differently across the different participant groups, leading to poorer model fit when they were all considered together. As the questionnaire aimed to distinguish those with RUCC from the other conditions based on cough sensations and triggers, the items that had an excellent fit for those with RUCC (rather than the group as a whole), were included in the final questionnaire. The mean and range of scores for each of the final 15 items (0 never–5 always) are presented in table 2, with TOPIC total scores ranging from 0 to 75.

**Table 2:** Median scores (IQR) for TOPIC items by diagnosis

TOPIC question median score (IQR)(N=140)	Diagnosis				
	RUCC(n=39)	ILD(n=38)	Asthma(n=45)	COPD(n=6)	Bronchiectasis(n=12)
My cough is triggered by certain foods	2.0 (0.0–4.0)	0.0 (0.0–1.0)	0.0 (0.0–0.0)	0.0 (0.0–0.0)	0.0 (0.0–0.0)
My cough is triggered by certain smells and odours	2.0 (0.0–4.0)	0.0 (0.0–2.0)	0.0 (0.0–2.0)	0.0 (0.0–2.8)	0.0 (0.0–0.0)
Swallowing triggers my cough	2.0 (0.0–2.0)	0.5 (0.5–1.0)	0.0 (0.0–1.0)	1.0 (0.0–1.3)	1.0 (0.0–1.8)
I cough after meals	2.0 (1.0–4.0)	1.0 (0.0–2.0)	0.0 (0.0–1.0)	0.0 (0.0–0.5)	0.0 (0.0–1.8)
I cough because I need to clear my throat	3.0 (2.0–5.0)	2.0 (1.0–4.0)	1.0 (1.0–2.0)	2.0 (0.8–2.3)	3.5 (3.0–4.8)
A dry throat triggers my cough	2.0 (1.0–4.0)	1.0 (0.0–2.0)	1.0 (0.0–2.0)	1.5 (0.8–3.3)	1.5 (1.0–3.8)
My cough is triggered by talking on the telephone	2.0 (1.0–4.0)	1.5 (0.0–2.0)	0.0 (0.0–1.0)	0.0 (0.0–1.5)	1.0 (0.0–3.8)
My cough is triggered by talking face to face with people I know	2.0 (0.0–2.0)	1.0 (0.0–2.0)	0.0 (0.0–0.0)	0.0 (0.0–0.8)	0.5 (0.0–2.8)
Coughing makes me feel like I’m choking	2.0 (1.0–4.0)	1.0 (1.0–2.0)	1.0 (0.0–1.0)	2.0 (0.0–3.5)	1.0 (1.0–2.0)
My cough makes me feel embarrassed	4.0 (2.0–5.0)	2.0 (1.0–4.0)	0.0 (0.0–1.0)	1.0 (0.0–2.8)	1.0 (1.0–4.5)
I find my cough annoying	5.0 (4.0–5.0)	4.0 (2.0–5.0)	1.0 (0.0–2.5)	2.5 (1.0–5.0)	2.5 (1.3–4.8)
My cough gives me a headache/pain in my head	1.0 (0.0–3.0)	0.5 (0.0–2.0)	0.0 (0.0–1.0)	0.5 (0.5–2.3)	0.0 (0.0–2.5)
I cough so violently that I retch	2.0 (1.0–3.0)	1.0 (0.0–2.0)	0.0 (0.0–1.0)	0.5 (0.0–2.3)	1.0 (0.0–1.8)
Coughing causes me to feel pressure in my head	2.0 (0.0–3.0)	0.0 (0.0–1.3)	0.0 (0.0–1.0)	0.5 (0.0–1.3)	0.0 (0.0–1.5)
When I cough I cannot control it	4.0 (3.0–5.0)	3.0 (1.0–4.0)	1.0 (0.5–2.0)	3.5 (1.5–5.0)	3.0 (1.3–4.0)
Median total TOPIC score	37.0	24.5	7.0	18.5	20.0

COPD, chronic obstructive pulmonary disease; ILD, interstitial lung disease; RUCC, refractory/unexplained chronic cough; TOPIC, The Sensations and Triggers Provoking Cough

**Figure 2:**
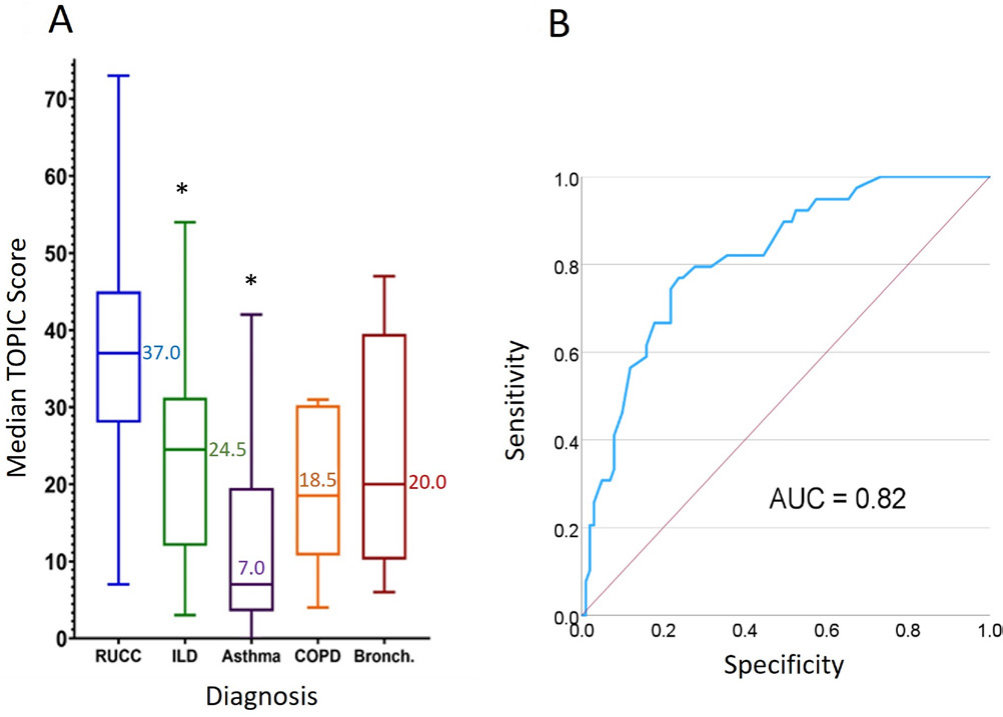
(**A**) Median TOPIC total scores (IQR) n=140 TOPIC completers. (**B**) ROC curve: TOPIC as a predictor of RUCC versus chronic cough in other groups (ILD, asthma, COPD and bronchiectasis). AUC, area under the curve; COPD, chronic obstructive pulmonary disease; ILD, interstitial lung disease; RUCC, refractory/unexplained chronic cough; TOPIC, The Sensations and Triggers Provoking Cough. *Significant difference in total TOPIC score versus RUCC (p≤0.05)

Median total TOPIC score was significantly higher in RUCC (37.0 (IQR 28.0–45.0)) vs ILD (24.5 (IQR 12.0–31.3), p=0.009) and asthma (7.0 (IQR 3.5–19.5), p<0.001), but not bronchiectasis (20.0 (IQR 10.3–39.5), p=0.318) or COPD (18.5 (IQR 10.8–30.4), p=0.238; figure 2A) where sample sizes were smaller. Figure 2B shows the ROC curve comparing the RUCC group versus non-RUCC cohorts (ILD, asthma, COPD and bronchiectasis), where the area under the curve is 0.82. This suggests that for 82% of cases, participants with RUCC would have a higher score than those with chronic cough associated with ILD, asthma, COPD or bronchiectasis.

**Figure 3:**
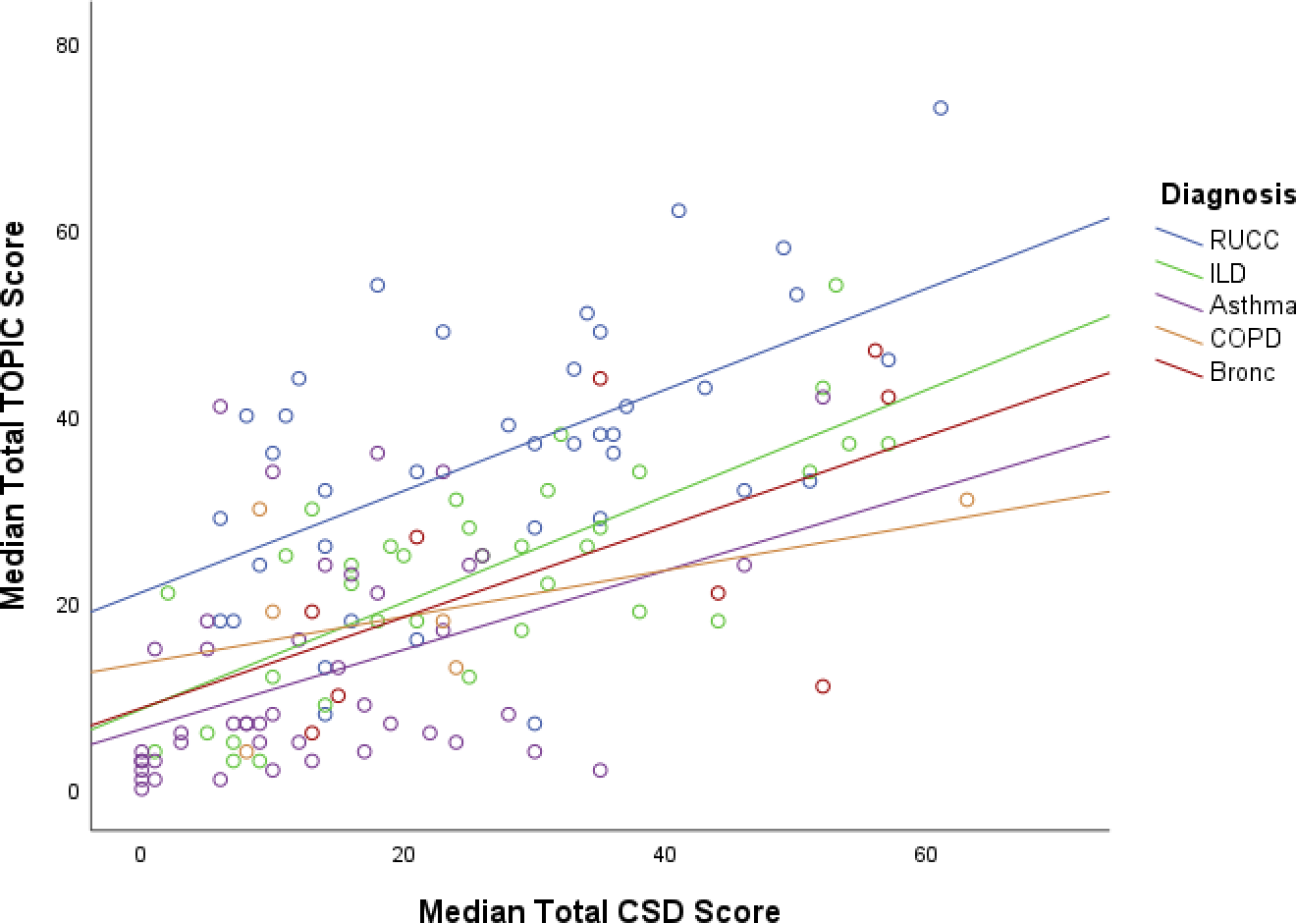
Scatterplot of total TOPIC scores and total Cough Severity Diary scores. Bronc, bronchiectasis; COPD, chronic obstructive pulmonary disease; ILD, interstitial lung disease; RUCC, refractory or unexplained chronic cough; TOPIC, The Sensations and Triggers Provoking Cough.

There was no significant difference in median CSD score between RUCC (30.00 (IQR 14.00–36.25)) and ILD (25.00 (IQR 13.00–35.00), p=0.290), bronchiectasis (35.00 (IQR 14.00–54.00) p=0.559) or COPD (16.50 (IQR 8.75–33.75), p=0.079); however, there was a significant difference versus asthma (10.00 (IQR 4.00–20.50), p=0.006). TOPIC total scores significantly correlated with the CSD total score (r=0.63, p<0.001; figure 3), SGRQ total score (r=0.47, p<0.001; online supplemental figure 2S) and subscales (symptoms r=0.44, p<0.001; activity r=0.31, p<0.001; impacts r=0.54, p<0.001). The TOPIC trigger subscales were also significantly correlated with all the other questionnaires, with the CSD total score (r=0.50, p<0.001), SGRQ total score (r=0.36, p<0.001) and subscales (symptom r=0.36, p<0.001; activity r=0.27, p=0.001; impacts r=−0.38, p<0.001). The TOPIC sensation subscale score was significantly correlated with all the other questionnaires, with the CSD total score (r=0.67), SGRQ total (r=0.48) and subscales (symptom r=0.45; activity r=0.36; impacts r=−0.52).

Test–retest reliability was assessed for the 30 participants who completed the TOPIC questionnaire 5–7 days again after initial completion and reported ‘about the same’ cough symptoms on the GRoC Scale. Test–retest reliability for the total scale demonstrated excellent repeatability (r=0.90), and the trigger and sensations subscales demonstrated good repeatability (r=0.87 and r=0.85, respectively). The TOPIC questionnaire demonstrated excellent internal consistency (α=0.92).

Eigenvalues indicate the variance explained by each questionnaire item; therefore, identifying the items most ‘important’ to the questionnaires underlying structure. Exploratory factor analysis presented four domains with acceptable eigenvalues: (1) talking triggers (items 7 and 8 (α=0.78)) and frustrations (items 11 and 15 (α=0.78)); (2) food and olfactory triggers (items 1–4 (α=0.74)); (3) distressing sensations (items 9, 10 and 12 (α=0.77))’ and (4) ‘sensations related to head’ (items 13 and 14 (α=0.91)) (online supplemental table 5S).

## Discussion

The TOPIC questionnaire has been developed to characterise the triggers and sensations of cough in RUCC, from cough in other respiratory diseases. Questionnaire items were generated from interviews across six chronic cough aetiologies (stage I/II), where the most frequent and articulate descriptors of cough comprised the draft TOPIC questionnaire. Following completion of the draft TOPIC questionnaire by participants with RUCC, asthma, ILD, COPD and bronchiectasis (stage III), post hoc hierarchical and Rasch analyses facilitated the removal of items with poor ‘fit’ for RUCC; refining the 49-item draft questionnaire to the 15-item TOPIC questionnaire. TOPIC has good psychometric properties with no redundant items, excellent internal consistency and test–retest reliability. The TOPIC questionnaire shows promise to characterise the sensations and triggers of coughing experienced by patients with RUCC.

The key sensations, triggers and secretion descriptors of cough identified in stages I and II interviews are consistent with those identified in previous patient reports and focus group discussions in chronic cough.^17 38^ Hierarchical and Rasch analysis of the TOPIC draft questionnaire identified items experienced less often in the RUCC cohort; this included emotional triggers, irritation in the throat, and vomiting following cough. Three of the seven phonation cough triggers—talking in a group, talking to unfamiliar people and laughing—were removed for the same reason, further elucidating the role of phonation triggers in this group. Of the remaining items with ‘fit’ for RUCC, four domains were identified including ‘talking triggers and frustrations’, ‘food and olfactory triggers’, ‘distressing sensations’ and ‘sensations related to the head’; all of which have previously been reported as disease burden in RUCC.^1438^,^41^ The inclusion of both ‘sensations relating to the head’ questionnaire items headache and pressure in the head) was deemed essential for maintaining the questionnaire’s integrity, as the removal of either significantly affected the model’s fit. Although these items are correlated, their introduction into the Rasch model did not indicate a high level of local dependence (>0.07) and there is no agreed level when removing items.^35^

The ROC curve analysis indicated high sensitivity and specificity in identifying RUCC versus non-RUCC participants using TOPIC. As some sensations and triggers can be ubiquitously reported across respiratory conditions^12^ and subgroups of patients with respiratory disease may also have cough hypersensitivity, some overlap between RUCC and non-RUCC scores is expected, despite the removal of items scored highly by non-RUCC cohorts.

Previous works have demonstrated some value in using cough sensations and triggers to characterise subgroups within chronic cough. In an unselected chronic cough cohort attending a cough clinic, Hilton *et al* identified clusters of patients defined by the type and frequency of cough-related sensations and triggers.^12^ Similarly, Won *et al* found that talking, food and olfactory triggers were reported more frequently by patients with UCC versus those with unselected chronic cough using a Korean translation of the Cough Hypersensitivity Questionnaire, however, the development of this questionnaire is unpublished.^14^ The HARQ assesses symptoms thought to be associated with airway reflux and includes some questions about sensations of postnasal drip and a tickle/lump in the throat alongside some cough triggers. It has been shown to discriminate patients with chronic cough from healthy controls without a chronic cough.^19^ However, to the best of our knowledge, none of these questionnaires have been specifically developed to characterise the sensations and triggers of coughing in RUCC, from that associated with respiratory diseases.

Finally, it is interesting that the total TOPIC score was moderately correlated with the total CSD score, suggesting that cough sensations and triggers are related to perceived cough severity. Moderate correlations were also seen with total SGRQ score across all groups. Interestingly, SGRQ not only correlated with the sensation subscale of TOPIC (which was expected due to overlapping sensation items between the questionnaires) but also the TOPIC trigger subscale, despite the SGRQ having no cough trigger items. This suggests that the frequency of cough sensations and triggers as measured by TOPIC correlates with impaired health and perceived well-being in patients with chronic cough. Development of novel tools such as TOPIC may aid the clinical characterisation and identification of cough in patients with RUCC, compared with cough in other conditions.

### Limitations

While this was an unselected cohort, participants were all English-speaking, predominantly white individuals recruited from one tertiary site, where findings may not be broadly applicable to other settings or populations. Differences between the total TOPIC score in the RUCC cohort versus the COPD and bronchiectasis participants did not reach significance, most likely due to the much smaller sample sizes for both groups. 36 participants did not fully complete the draft TOPIC questionnaire. While reasons for this are not reported, the reduction in the questionnaire items and optimisation of formatting in the final TOPIC questionnaire (sensations and triggers on separate pages, 15 items to complete instead of 49) should promote full completion of the final version. Finally, the TOPIC has only been subjected to preliminary validity testing and requires further assessment against further cough measures.

## Conclusions

This work has characterised the distinct sensations and triggers of cough in RUCC compared with cough in other conditions. Currently, there are no structured or validated measures to capture the sensations and triggers associated with coughing. Future work will involve the application of TOPIC in multiple cough clinics, investigating relationships with other measures of cough, responses to treatment and the ability of the questionnaire to identify patients with RUCC.

## supplementary material

10.1136/bmjresp-2024-002430 online supplemental file 1

10.1136/bmjresp-2024-002430 online supplemental file 2

## Data Availability

Data are available on reasonable request.

## References

[1] Schappert SM, Burt CW (2006). Ambulatory care visits to physician offices, hospital outpatient departments, and emergency departments: United States, 2001-02. Vital Health Stat 13.

[2] Song W-J, Chang Y-S, Faruqi S (2015). The global epidemiology of chronic cough in adults: a systematic review and meta-analysis. Eur Respir J.

[3] Young EC, Smith JA (2010). Quality of life in patients with chronic cough. Ther Adv Respir Dis.

[4] Chamberlain SAF, Garrod R, Douiri A (2015). The impact of chronic cough: a cross-sectional European survey. Lung.

[5] Al-Sheklly B, Satia I, Badri H (2018). P5 Prevalence of refractory chronic cough in a tertiary cough clinic. Thorax.

[6] Morice AH, Millqvist E, Bieksiene K (2020). ERS guidelines on the diagnosis and treatment of chronic cough in adults and children. Eur Respir J.

[7] Gibson P, Wang G, McGarvey L (2016). Treatment of unexplained chronic cough: CHEST guideline and expert panel report. Chest.

[8] Good JT, Rollins DR, Kolakowski CA (2018). New insights in the diagnosis of chronic refractory cough. Respir Med.

[9] Smith JA, Woodcock A (2016). Chronic cough. N Engl J Med.

[10] Morice AH, Millqvist E, Belvisi MG (2014). Expert opinion on the cough hypersensitivity syndrome in respiratory medicine. Eur Respir J.

[11] Everett CF, Kastelik JA, Thompson RH (2007). Chronic persistent cough in the community: a questionnaire survey. Cough.

[12] Hilton E, Marsden P, Thurston A (2015). Clinical features of the urge-to-cough in patients with chronic cough. Respir Med.

[13] Vertigan AE, Gibson PG (2011). Chronic refractory cough as a sensory neuropathy: evidence from a reinterpretation of cough triggers. J Voice.

[14] Won H-K, Kang S-Y, Kang Y (2019). Cough-related laryngeal sensations and triggers in adults with chronic cough: symptom profile and impact. Allergy Asthma Immunol Res.

[15] Solomon A, Cho PP, Fletcher H (2017). P103 The urge to cough in COPD.

[16] Kum E, Guyatt GH, Devji T (2021). Cough symptom severity in patients with refractory or unexplained chronic cough: a systematic survey and conceptual framework. Eur Respir Rev.

[17] Kum E, Guyatt GH, Munoz C (2022). Assessing cough symptom severity in refractory or unexplained chronic cough: findings from patient focus groups and an international expert panel. ERJ Open Res.

[18] Vertigan AE, Bone SL, Gibson PG (2014). Development and validation of the Newcastle laryngeal hypersensitivity questionnaire. Cough.

[19] Morice AH, Faruqi S, Wright CE (2011). Cough hypersensitivity syndrome: a distinct clinical entity. Lung.

[20] La-Crette J, Lee KK, Chamberlain S (2012). P150 The development of a Cough Hypersensitivity Questionnaire (CHQ). Thorax.

[21] Kum E, Guyatt G, Abdulqawi R (2024). Development of the McMaster Cough Severity Questionnaire (MCSQ): a cough severity instrument for patients with chronic cough (abstract). Am J Respir Crit Care Med.

[22] Yorke J, Moosavi SH, Shuldham C (2010). Quantification of dyspnoea using descriptors: development and initial testing of the Dyspnoea-12. Thorax.

[23] Elm E von, Altman DG, Egger M (2007). Strengthening the reporting of observational studies in epidemiology (STROBE) statement: guidelines for reporting observational studies. BMJ.

[24] Vernon M, Leidy NK, Nacson A (2009). Measuring cough severity: perspectives from the literature and from patients with chronic cough. Cough.

[25] Davenport PW, Sapienza C, Bolser D (2002). Psychophysical assessment of the urge-to-cough. Eur Respir Rev.

[26] Solari F, Sunger K, Yorke J (2011). S143 The sensations provoking cough: a qualitative investigation. Thorax.

[27] Wilson Van Voorhis CR, Morgan BL (2007). Understanding power and rules of thumb for determining sample sizes. TQMP.

[28] Jones PW, Quirk FH, Baveystock CM (1992). A self-complete measure of health status for chronic airflow limitation. The St. George’s respiratory questionnaire. Am Rev Respir Dis.

[29] Jones PW, Quirk FH, Baveystock CM (1991). The St George’s respiratory questionnaire. Respir Med.

[30] Vernon M, Kline Leidy N, Nacson A (2010). Measuring cough severity: development and pilot testing of a new seven-item cough severity patient-reported outcome measure. Ther Adv Respir Dis.

[31] Martin Nguyen A, Bacci E, Dicpinigaitis P (2020). Quantitative measurement properties and score interpretation of the cough severity diary in patients with chronic cough. Ther Adv Respir Dis.

[32] Galgani S, Boone L, Wingfield-Digby J (2022). S18 describing the triggers and sensations associated with coughing across different disease groups. Thorax.

[33] Digby JW, Sawyer C, Galgani S (2023). A novel questionnaire describing sensations and triggers associated with refractory and unexplained chronic cough. Eur Respir Soc.

[34] Miles MB, Huberman AM (1994). Qualitative Data Analysis: An Expanded Sourcebook.

[35] Linacre JM (1994). Rasch Measurement Transactions.

[36] Hair J, Black W, Babin B (2009). Multivariate Data Analysis.

[37] Field A (2013). Discovering Statistics Using IBM SPSS.

[38] Hirons B, Rhatigan K, Kesavan H (2024). Qualitative assessment of sensations and triggers in chronic cough. ERJ Open Res.

[39] Ueda N, Yakushiji A, Schelfhout J (2022). Impact of refractory and unexplained chronic cough on disease burden: a qualitative study. BMC Pulm Med.

[40] Sundar KM, Stark AC, Hu N (2021). Is laryngeal hypersensitivity the basis of unexplained or refractory chronic cough?. ERJ Open Res.

[41] Morice A, Birring S, Dicpinigaitis P (2021). COUGH triggers and symptoms among patients with refractory or unexplained chronic COUGH in two Phase 3 Trials of the P2X3 receptor antagonist Gefapixant (COUGH-1 and COUGH-2). J Allergy Clin Immunol.

