# Supplementary material for: Development and validation of a novel questionnaire to describe and assess sensations and triggers associated with refractory and unexplained chronic cough
Source: BMJ Open Respir Res. 2024 Aug 13;11(1):e002430. doi: 10.1136/bmjresp-2024-002430 (PMC11331982; doi:10.1136/bmjresp-2024-002430)

Supplement

Figure 1S: Sensations Provoking Cough Interview Guide for the TOPIC Study (Stage I)

The interview guide was developed following a literature review of studies exploring people's experience and descriptions of cough and incorporating the experience of clinicians delivering local cough services.

<u>Sensations Provoking Cough TOPIC Guide</u>

- Do you feel an urge to cough?
- How would you describe this feeling?
- Do you produce any sputum? And if you do how much?
- Is your urge to cough painful?
- How intense is the urge to cough feeling?
- Is the intensity or feeling of the urge to cough different in the day or night time?
- How often do you get the urge to cough?
- Does anything specific trigger your urge to cough?
- If you get the urge to cough do you always cough afterwards?
- Does coughing relieve the urge?
- Does anything else relieve the urge to cough?
- How does the urge to cough make you feel?
- Where is the feeling of the urge to cough?
    - Central, lateral, bilateral
    - Localised or diffuse
- Is there anything else you would like to discuss about your cough?

Table 1S: Key Descriptors of Cough Identified from Stage I of the TOPIC Study

Content analysis of the most frequently observed words or phrases from the audio transcripts of stage I participant interviews identified 18 key descriptors of cough across three themes: sensations, triggers, and secretions.

Secretions	Triggers	Sensations
Sputum	Eating	Urge to Cough
Phlegm	Talking	Need to Cough
Catarrh	Lying Flat	Desire to Cough
Mucus	Specific smells and odours	Tickle
Spit	Specific foods	Irritation
		Itch
		Choking
		Crackle

Table 2S: Draft 'The triggers and sensations provoking cough (TOPIC)' Questionnaire.

The 49 most frequent and articulated descriptions of cough sensations and triggers from Stage I and Stage II of the TOPIC Study compiled the draft TOPIC questionnaire.

TRIGGERS	
In this section of the questionnaire we would like you to think about different triggers for your cough. For each statement below, please tick the answer that best describes your recent experience of cough.	
1)	An irritation in my chest has triggered my cough
2)	An irritation in my throat triggers my cough
3)	A tickle in my throat triggers my cough
4)	There are no specific triggers for my cough
5)	My cough is unpredictable
6)	My cough is triggered by certain foods
7)	My cough is triggered by certain smells and odours
8)	Swallowing triggers my cough
9)	I cough after meals
10)	A change in air temperature triggers my cough
11)	Changes in the weather triggers my cough (e.g. changes in humidity)
12)	A high pollen count triggers my cough
13)	Poor air quality (e.g. pollution, smoky atmospheres) triggers my cough
14)	Lying flat makes me cough
15)	Exertion or exercise makes me cough
16)	Deep breathing makes me cough
17)	Breathlessness causes me to cough
18)	Phlegm (sputum) triggers my cough
19)	I cough because I need to clear my throat
20)	A dry throat triggers my cough
21)	My cough is triggered by talking on the telephone
22)	My cough is triggered by talking face to face with people I know
23)	My cough is triggered by talking face to face with unfamiliar people
24)	My cough is triggered by talking in a group situation
25)	Being anxious causes me to cough

26) Being anxious before talking (in social or formal situations) makes me cough

27) Laughing triggers my cough

28) Crying triggers my cough

29) Happy emotions trigger my cough (e.g. happiness, joy)

30) Sad emotions (e.g. sorrow, fear)

31) My cough is triggered by stressful situations

SENSATIONS

In this section we would like you to think about the sensations you may experience when coughing and how this makes you feel. For each statement below, please tick the answer that best describes your recent experience of cough.

32) Coughing makes me feel like I'm choking

33) When I'm coughing I feel like I can't get my breath

34) I find my cough unpleasant

35) My cough is unpleasant for other people (e.g. socially, at work)

36) My cough makes me feel embarrassed

37) I find my cough annoying

38) My cough is annoying for others

39) My cough make me feel uncomfortable

40) I cough so violently that I retch

41) I cough so violently that I vomit

42) Coughing makes me breathless

43) Coughing prevents me from getting air into my lungs

44) Coughing makes me feel exhausted

45) My cough gives me a headache/pain in my head

46) Coughing causes me to feel pressure in my head

47) My chest feels like it tightens after coughing

48) Coughing causes muscular pain (e.g. back, neck, chest)

49) When I cough I cannot control it

Table 3S: Reason for item removal for draft TOPIC Trigger items

Floor effect was achieved when ≥50.0% of participants scored 0 or 1 and Ceiling effect was achieved when ≥50.0% of participant scored 4 or 5.

1 ≥50 % of RUCC participants

2 ≥50 % of COPD, Bronchiectasis, Asthma, ILD participants

3 ≥50 % of RUCC, COPD, Bronchiectasis, Asthma, ILD participants

Abbreviations COPD, chronic obstructive pulmonary disease; ILD, interstitial lung disease.

	Draft TOPIC item	Floor[1]	Ceiling[2]	Rasch
1	An irritation in my chest has triggered my cough	X[1]		
2	An irritation in my throat triggers my cough		x[1]	
3	A tickle in my throat triggers my cough		x[1]	
4	There are no specific triggers for my cough	X[3]		
5	My cough is unpredictable		x[3]	
23	My cough is triggered by talking face to face with unfamiliar people	x[3]		
24	My cough is triggered by talking in a group situation	x[3]		
25	Being anxious causes me to cough	x[3]		
26	Being anxious before talking (in social or formal situations) makes me cough	x[3]		
27	Laughing triggers my cough	x[3]		
28	Crying triggers my cough	x[3]		
29	Happy emotions trigger my cough (e.g. happiness, joy)	x[3]		
30	Sad emotions (e.g. sorrow, fear)	x[3]		
6	My cough is triggered by certain foods			
7	My cough is triggered by certain smells and odours			
8	Swallowing triggers my cough			
9	I cough after meals			
10	A change in air temperature triggers my cough			x
11	Changes in the weather triggers my cough (e.g. changes in humidity)			x
12	A high pollen count triggers my cough			x
13	Poor air quality (e.g. pollution, smoky atmospheres) triggers my cough			x
14	Lying flat makes me cough			x
15	Exertion or exercise makes me cough			x
16	Deep breathing makes me cough			x
17	Breathlessness causes me to cough			x
18	Phlegm (sputum) triggers my cough			x
19	I cough because I need to clear my throat			
20	A dry throat triggers my cough			
21	My cough is triggered by talking on the telephone			
22	My cough is triggered by talking face to face with people I know (changed to talking)			
31	My cough is triggered by stressful situations			x
Number of items removed		10	3	10

Table 4S: Reason for item removal for Draft TOPIC Sensation items.

Floor effect was achieved when ≥50.0% of participants scored 0 or 1 and Ceiling effect was achieved when ≥50.0% of participant scored 4 or 5.

1 ≥50 % of RUCC participants

2 ≥50 % of COPD, Bronchiectasis, Asthma, ILD participants

3 ≥50 % of RUCC, COPD, Bronchiectasis, Asthma, ILD participants

Draft TOPIC Item		Floor	Ceiling	Rasch
34	My cough is unpleasant		X[3]	
35	My cough is unpleasant for other people (e.g. socially, at work		x[1]	
38	My cough is annoying for others		x[3]	
39	My cough make me feel uncomfortable		X[3]	
41	I cough so violently that I vomit	x[3]		
32	coughing makes me feel like I'm choking			
33	When I'm coughing I feel like I can't get my breath			x
36	My cough makes me feel embarrassed			
37	I find my cough annoying			
40	I cough so violently that I retch/vomit			
42	Coughing makes me breathless			x
43	Coughing prevents me from getting air into my lungs			x
44	Coughing makes me feel exhausted			x
45	My cough gives me a headache/ muscular pain			
46	Coughing causes me to feel pressure in my head			
47	My chest feels like it tightens after coughing			x
48	Coughing causes muscular pain (e.g. back, neck, chest)			x
49	When I cough I cannot control it			
Number of items removed		1	4	6

Table 5S Eigen values from the Principal Component Analyses

Extraction Method: Principal Axis Factoring. Rotation Method: Varimax with Kaiser Normalization.

[a]Rotation converged in 9 iterations

Rotated Factor Matrix[a]

TOPIC Items	Factor			
	Talking triggers and Frustrations 1	Food and Olfactory Triggers 2	Distressing Symptoms 3	Sensations related to the head 4
Q15) When I cough I cannot control it	.650			
Q11) I find my cough annoying	.644			
Q7) My cough is triggered by talking on the telephone	.639			
Q8) My cough is triggered by talking face to face with people I know	.513			
Q1) My cough is triggered by certain foods		.837		
Q4) I cough after meals		.603		
Q3) Swallowing triggers my cough		.501		
Q2) My cough is triggered by certain smells and odours		.541	.621	
Q12) I cough so violently that I retch			.647	
Q9) Coughing makes me feel like I'm choking			.627	
Q10) My cough makes me feel embarrassed	.526		.555	
Q14) Coughing causes me to feel pressure in my head				.885
Q13) My cough gives me a headache/pain in my head				.885
Q5) I cough because I need to clear my throat				
Q6) A dry throat triggers my cough				

Figure 2S Scatterplot of Total TOPIC Scores and total SGRQ scores. Abbreviations Bronc, bronchiectasis; COPD, chronic obstructive pulmonary disease; ILD, interstitial lung disease; RUCC, refractory or unexplained chronic cough; SGRQ, St. George's Respiratory Questionnaire.

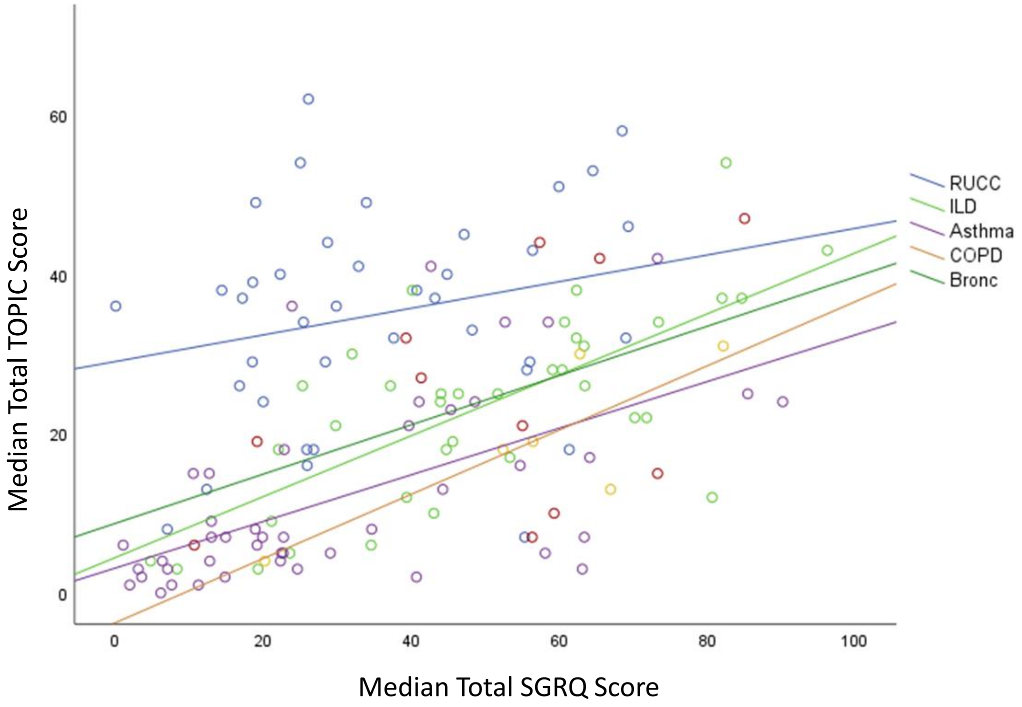

Supplement: online supplemental file 1 [file bmjresp-11-1-s001.pdf]
